# Nanometer-long Ge-imogolite nanotubes cause sustained lung inflammation and fibrosis in rats

**DOI:** 10.1186/s12989-014-0067-z

**Published:** 2014-12-14

**Authors:** Sybille van den Brule, Emilie Beckers, Perrine Chaurand, Wei Liu, Saloua Ibouraadaten, Mihaly Palmai-Pallag, Francine Uwambayinema, Yousof Yakoub, Astrid Avellan, Clément Levard, Vincent Haufroid, Etienne Marbaix, Antoine Thill, Dominique Lison, Jérôme Rose

**Affiliations:** Louvain centre for Toxicology and Applied Pharmacology, Université catholique de Louvain, Avenue E. Mounier 52 - bte B1.52.12, 1200 Brussels, Belgium; CEREGE, Aix Marseille Université, CNRS, IRD, UM34, UMR 7330, Europole de l’arbois - BP 80, 13545 Aix en Provence, France; De Duve Institute, Université catholique de Louvain, Avenue Hippocrate 75 - bte B1.75.02, 1200 Brussels, Belgium; Laboratoire Interdisciplinaire sur l’Organisation Nanométrique et Supramoléculaire, CEA Saclay, 91191 Gif sur Yvette, France; iCEINT, International Consortium for the Environmental Implications of Nanotechnology, CNRS, Duke University, 13545 Aix en Provence, France; Department of Clinical Chemistry, Cliniques Universitaires Saint-Luc, Université catholique de Louvain, Avenue Hippocrate 10, 1200 Brussels, Belgium

**Keywords:** Lung, Inflammation, Fibrosis, Genotoxicity, Nanomaterial, Fibre, High aspect ratio, Biopersistence

## Abstract

**Background:**

Ge-imogolites are short aluminogermanate tubular nanomaterials with attractive prospected industrial applications. In view of their nano-scale dimensions and high aspect ratio, they should be examined for their potential to cause respiratory toxicity. Here, we evaluated the respiratory biopersistence and lung toxicity of 2 samples of nanometer-long Ge-imogolites.

**Methods:**

Rats were intra-tracheally instilled with single wall (SW, 70 nm length) or double wall (DW, 62 nm length) Ge-imogolites (0.02-2 mg/rat), as well as with crocidolite and the hard metal particles WC-Co, as positive controls. The biopersistence of Ge-imogolites and their localization in the lung were assessed by ICP-MS, X-ray fluorescence, absorption spectroscopy and computed micro-tomography. Acute inflammation and genotoxicity (micronuclei in isolated type II pneumocytes) was assessed 3 d post-exposure; chronic inflammation and fibrosis after 2 m.

**Results:**

Cytotoxic and inflammatory responses were shown in bronchoalveolar lavage 3 d after instillation with Ge-imogolites. Sixty days after exposure, a persistent dose-dependent inflammation was still observed. Total lung collagen, reflected by hydroxyproline lung content, was increased after SW and DW Ge-imogolites. Histology revealed lung fibre reorganization and accumulation in granulomas with epithelioid cells and foamy macrophages and thickening of the alveolar walls. Overall, the inflammatory and fibrotic responses induced by SW and DW Ge-imogolites were more severe (on a mass dose basis) than those induced by crocidolite. A persistent fraction of Ge-imogolites (15% of initial dose) was mostly detected as intact structures in rat lungs 2 m after instillation and was localized in fibrotic alveolar areas. In vivo induction of micronuclei was significantly increased 3 d after SW and DW Ge-imogolite instillation at non-inflammatory doses, indicating the contribution of primary genotoxicity.

**Conclusions:**

We showed that nm-long Ge-imogolites persist in the lung and promote genotoxicity, sustained inflammation and fibrosis, indicating that short high aspect ratio nanomaterials should not be considered as innocuous materials. Our data also suggest that Ge-imogolite structure and external surface determine their toxic activity.

**Electronic supplementary material:**

The online version of this article (doi:10.1186/s12989-014-0067-z) contains supplementary material, which is available to authorized users.

## Background

The health hazards of nanomaterials (NM) are subject to intense research efforts, and high aspect ratio NM (HARN, including nanotubes) have received particular attention because of their potential to cause adverse effects on the respiratory tract similar to asbestos fibres, including inflammation, fibrosis, genotoxicity, lung cancer and mesothelioma. HARN (carbon nanotubes CNT and silver nanowires) longer than 15 or 5 μm persist for extended periods in the lung or pleura, respectively, and induce local inflammation [1-3] and fibrosis [1,2,4-7], indicating that HARN length is a crucial determinant of their toxicity [1,8,9].

Imogolites occur naturally as single wall (SW) tubular aluminosilicates that can be classified as HARN and display some structure and composition similarities to asbestos. Their length can vary from a few nm to several μm and their diameter is in the nm range. A wide range of industrial applications are in prospect for imogolites because of their electrical, mechanical and chemical properties, including their use as catalyst support and gas storage materials [10]. Imogolites can also be synthetized in a controlled manner as SW or double wall (DW) nanotubes, and with an improved yield by substituting Si by Ge [11,12].

In the present study, we examined the lung toxicity of nanometer-long SW and DW Ge-substituted imogolites. We report that Ge-imogolite nanotubes induce severe pulmonary inflammation, fibrosis and genotoxicity in rats, indicating that short HARN also represent a potential risk for human health. We additionally used, for the first time, 2D and 3D X-ray imaging techniques to demonstrate that intact Ge-imogolite structures persist in the lung tissue.

## Results and discussion

Samples of SW and DW Ge-substituted imogolites were synthesized as concentrated suspensions and characterized as described below (Table 1). Outer wall diameter and number of walls were measured by small angles X-ray scattering (SAXS), tube length distribution by atomic force microscopy (AFM) and concentration in suspension (g Ge-imogolites/l) by inductively coupled plasma-mass spectrometry (ICP-MS, see Additional file 1: Figure S1 and Additional file 2). The mean length, diameter and theoretical external surface of SW and DW Ge-imogolites were similar and the number of SW nanotubes per g of Ge-imogolites was 1.54 higher than for DW (Table 1). Total external surface per g SW Ge-imogolites (specific external surface area) was accordingly higher than for DW. Both size distributions were broad, ranging from very short (below 40 nm) to short tubes (100–200 nm), though SW tube distribution was multimodal and much wider than for DW, in accordance with very broad and dissymmetric length distributions reported previously [13,14].

**Table 1:** **Physico-chemical characteristics of Ge-imogolites, crocidolite and WC-Co**

	**SW imogolites**	**DW imogolites**	**Crocidolite**	**WC-Co**
Diameter	3.8 nm^a^	4.3 nm^a^	330 nm ± 2.1^b^	2 μm^c^
Length	70 nm ± 31^d^	62 nm ± 19^d^	2.5 μm ± 2^b^	-
External surface	837.5 nm^2^/tube^e^	843.74 nm^2^/tube^e^	8 m^2^/g^f^	1.76 m^2^/g^f^
Number of Ge/ring^g^	19	22 + 11	-	-
Number of ring/tube^g^	210	186	-	-
Mass per tube^h^	1.61 × 10^−18^ g	2.47 × 10^−18^ g	-	-
Concentrations				
g Ge/l^i^	2.3	2.2	-	-
g Ge-imogolites/l^j^	7.68	7.33		
tubes/g Ge-imogolites^k^	6.22 × 10^17^	4.05 × 10^17^		
nm^2^/g^l^	5.21 × 10^20^	3.41 × 10^20^		
m^2^/g	5.21 × 10^11^	3.41 × 10^11^		

^a^Outer wall diameter for imogolites measured by SAXS.

^b^Geometric mean ± standard deviation.

^c^ *d*_*50*_.

^d^Mean ± standard deviation measured by AFM.

^e^Theoretical external surface (S = π d L).

^f^Specific surface area [29,30].

^g^Based on SAXS curve modeling.

^h^Number of Ge/ring x number of ring/tube x Al_2_GeO_3_(OH)_4_ total MW (242.63 g/mol) / *N*_*A*_ (6.022 × 10^23^ tubes/mol).

^i^Concentrations of stock suspensions determined by ICP-MS.

^j^Imogolite concentrations were calculated based on the elementary composition of Ge-imogolites (Al_2_GeO_3_(OH)_4_, total MW = 242.63 g/mol and Ge MW = 72.64 g/mol).

^k^Calculated from the mass per tube.

^l^External surface area/g calculated from tubes/g Ge-imogolites and theoretical external surface.

**Figure 1:**
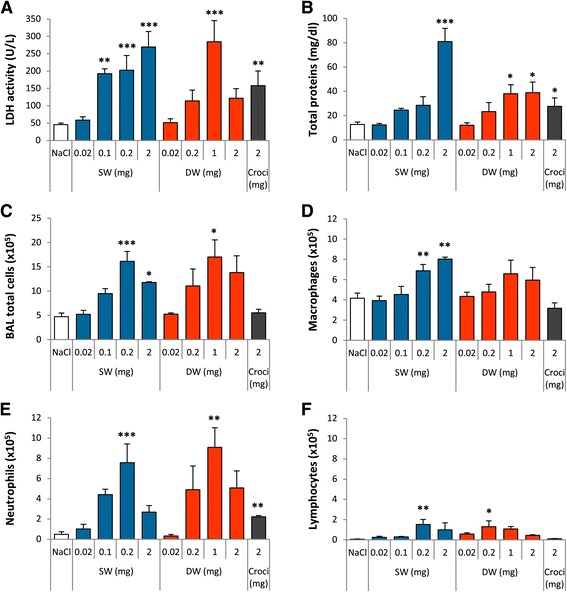
**Ge-imogolites induce a strong inflammation in rat lungs 3 d after intra-tracheal instillation.** Wistar rats were intra-tracheally instilled with NaCl (controls), 2 mg crocidolite, 0.02 to 2 mg SW and DW Ge-imogolites. Inflammation was investigated in the BAL after 3 d. **(A)** LDH activity and **(B)** total proteins measured in BALF. BAL number of **(C)** total cells, **(D)** macrophages, **(E)** neutrophils and **(F)** lymphocytes. *P < 0.05, **P < 0.01 and ***P < 0.001 relative to NaCl-treated rats (Dunnett multiple comparisons test between NaCl and Ge-imogolite-treated rats or t-test between NaCl and crocidolite-treated rats, n = 3–6, means ± SEM).

An intra-tracheal instillation model was used for this first in vivo study because Ge-imogolites were produced in suspension and in small amounts. Because respiratory toxicity of inhaled materials is often associated with their biopersistence, we first assessed the biopersistence of Ge-imogolites in rat lungs by measuring Ge content by ICP-MS directly after an intra-tracheal instillation of a non-inflammatory dose (0.02 mg Ge-imogolites, equivalent to 6 μg Ge/rat) as well as after 15 and 60 d. No significant inflammatory lung response was observed 3 d after instillation of this dose (Figure 1). Additional file 3: Figure S2 A and B show that 5 μg Ge were recovered from the lung immediately after instillation. After 15 d, approximately 60% of this initial Ge dose was measured in lungs and 15% after 60 d, indicating half-lives of 21 and 24 d, for SW and DW Ge-imogolites respectively, as determined by non-linear regression. Thus, Ge-imogolite lung clearance appeared to follow the usual retention time course reported for non-soluble inhaled particles [15] and some CNT [16,17]. In blood, Ge was detected 3 d after instillation of Ge-imogolites while very low amounts were found after 60 d (Additional file 3: Figure S2C). Low levels of Ge were measured in peripheral organs after 60 d (Additional file 3: Figure S2D).

**Figure 2:**
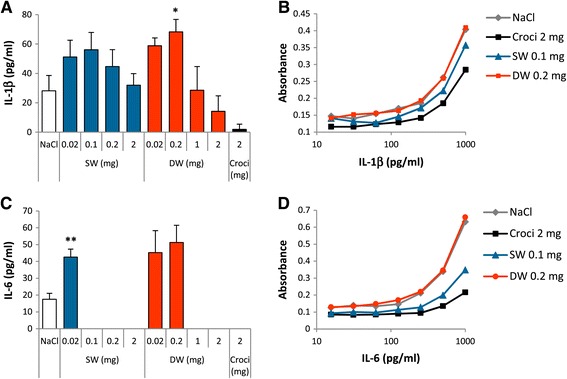
**Ge-imogolites induce inflammatory cytokine secretion in rat alveoli 3 d after intra-tracheal instillation.** Wistar rats were intra-tracheally instilled with NaCl (controls), 2 mg crocidolite, 0.02 to 2 mg SW and DW Ge-imogolites. Inflammatory cytokines were quantified by ELISA in the BALF after 3 d. BALF **(A)** IL-1β and **(C)** IL-6. **(B)** IL-1β and **(D)** IL-6 curves obtained from cytokine standards diluted with BALF from NaCl, 2 mg crocidolite, 0.1 mg SW and 0.2 mg DW Ge-imogolite-exposed rats. *P < 0.05 and **P < 0.01 relative to control rats (Dunnett multiple comparisons test between NaCl and Ge-imogolite-treated rats or t-test between NaCl and crocidolite-treated rats, n = 4, means ± SEM).

To assess the lung response, rats were intra-tracheally instilled with 0.02-2 mg SW or DW Ge-imogolites, a dose range selected from a recent study with CNT [4]. The acute pulmonary response was studied after 3 d in comparison to 2 mg crocidolite selected as a positive control (Figure 1). Cytotoxicity and alveolo-vascular integrity were assessed by measuring LDH activity released from damaged cells and total protein concentration in BALF, respectively. Compared to control rats (NaCl), these markers increased in a dose-dependent manner except at the highest dose tested. The highest dose suspensions (2 mg suspended in 300 μl NaCl) prepared for instillation were thicker than other suspensions. It is known that imogolites can occur naturally as gels and can form gels under certain conditions [18,19]. Thus, the instillation of 2 mg may have induced a weaker response for some parameters. SW and DW Ge-imogolites induced an inflammatory cell recruitment in the alveoli, mainly composed of macrophages and neutrophils whereas crocidolite mainly induced a neutrophil recruitment. A weak but significant lymphocyte increase also occurred 3 d after Ge-imogolite instillation. Prototypic pro-inflammatory cytokines interleukin (IL-) 1β, tumor necrosis factor (TNF) -α and IL-6 were measured in BALF by enzyme-linked immunosorbent assays (ELISA). While TNF-α levels were below the limit of detection in all samples, IL-1β and IL-6 increased at low Ge-imogolite doses but then dropped to zero or to the control level (Figure 2A and C). Since imogolites strongly interact with proteins [20], we suspected that they could interfere with the assay. Therefore, ELISAs were performed on protein standards diluted with BALF (see methods) and IL-1β and IL-6 standard curves were compared. Crocidolite and 0.1 mg SW Ge-imogolite BALF reduced IL-1β and IL-6 signals suggesting that the secretion of these cytokines was underestimated by the ELISA results (Figure 2B and D). DW Ge-imogolites at the instillation dose of 0.2 mg did not modify IL-1β and IL-6 signals, indicating that the cytokine increase observed at this dose is not an artifact. However, we cannot exclude the interference of the assay with DW Ge-imogolites at higher doses. Indeed, IL-6 standard signals were dose-dependently reduced in the presence of DW Ge-imogolites (data not shown), indicating that IL-1β and IL-6 might also be underestimated in DW Ge-imogolite BALF.

**Figure 3:**
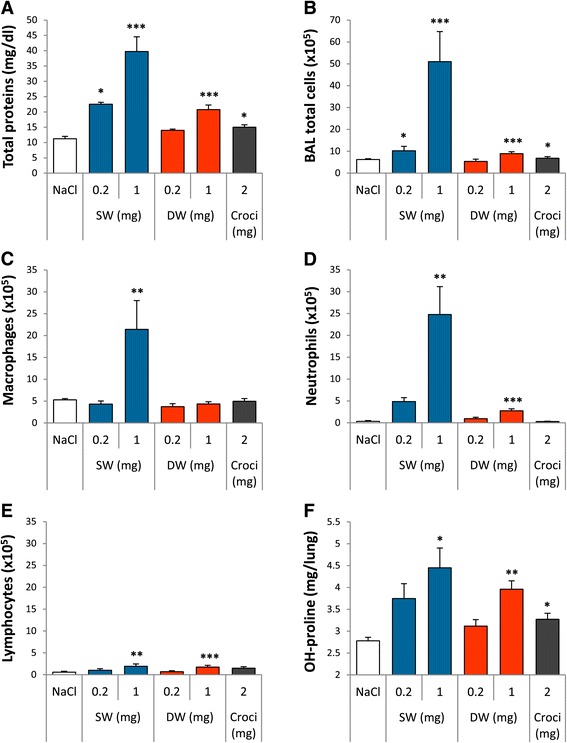
**Ge-imogolites induce a persistent inflammatory and fibrotic lung response.** Wistar rats were intra-tracheally instilled with NaCl (controls), 2 mg crocidolite, 0.2 and 1 mg SW and DW Ge-imogolites. Inflammatory and fibrotic parameters were measured after 60 d. **(A)** Total proteins in BALF. BAL number of **(B)** total cells, **(C)** macrophages, **(D)** neutrophils and **(E)** lymphocytes. **(F)** OH-proline lung content, measured in lung homogenates. *P < 0.05, **P < 0.01 and ***P < 0.001 relative to control rats (Dunnett multiple comparisons test between NaCl and Ge-imogolite-treated rats or t-test between NaCl and crocidolite-treated rats, n = 4, means ± SEM).

**Figure 4:**
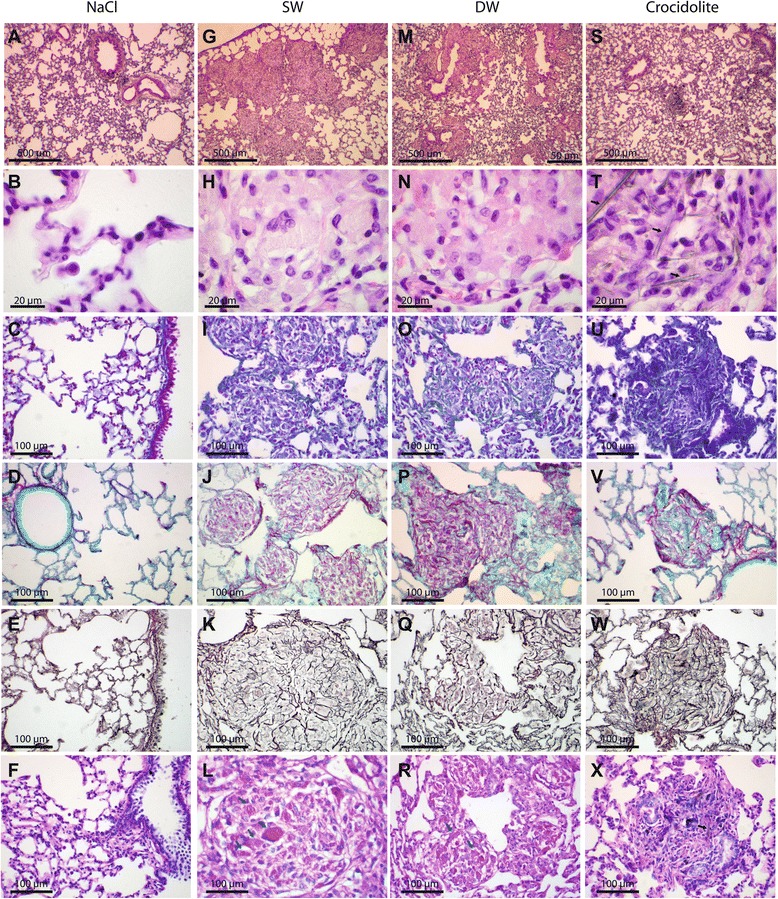
**Ge-imogolites induce a fibrotic lung response.** Wistar rats were intra-tracheally instilled with NaCl (controls), 2 mg crocidolite, 1 mg SW and DW Ge-imogolites. Lung histology was examined 60 d after exposure. **(A-F)** NaCl, **(G-L)** SW Ge-imogolites, **(M-R)** DW Ge-imogolites and **(S-X)** crocidolite. Sections stained with H&E **(A-B,G-H,M-N,S-T)**, Trichrome blue **(C,I,O,U)**, Sirius Red **(D,J,P,V)**, reticulin **(E,K,Q,W)**, PAS **(F,L,R,X)**. Black and blue arrows indicate asbestos fibres and glycoprotein-rich areas, respectively.

Chronic inflammatory and fibrotic lung responses in the lungs were assessed 60 d after instillation with 0.2 and 1 mg SW and DW Ge-imogolites per rat. Total proteins and BAL inflammatory cells remained elevated 60 d after instillation, the response after SW Ge-imogolites being more severe than after DW (Figure 3A and B). LDH data were very similar to total proteins (data not shown). BAL neutrophils and lymphocytes were increased after SW and DW Ge-imogolites at the dose of 1 mg, whereas macrophages were elevated only after 1 mg SW (Figure 3C to E). BALF levels of pro-inflammatory cytokines IL-6, IL-1β and TNF-α as well as fibrogenic growth factors transforming growth factor (TGF) -β, platelet-derived growth factor (PDGF) and osteopontin (OPN) were not increased 2 m after exposure to Ge-imogolites and even to crocidolite (data not shown). Total collagen accumulation assessed by measuring lung OH-proline content was increased dose-dependently after the administration of SW and DW Ge-imogolites as well as after 2 mg crocidolite (Figure 3F). Compared to control (NaCl), lung sections of animals treated with crocidolite stained with hematoxylin and eosin (H&E) showed inflammation, thicker alveolar walls and granulomas filled with asbestos fibres (Figure 4A and S). In SW and DW-treated lungs, numerous granulomas were observed in alveoli and near bronchioles, inflammation being more diffuse with DW (Figure 4G and M). In SW-treated lungs, macrophages were mostly concentrated in granulomas and lesions appeared more restricted whereas, in DW-treated lungs, macrophages and foam cells were also found in the alveolar spaces. Although alveolar spaces and walls outside nodules seemed better preserved in SW-treated lungs compared to DW, we observed similar surface areas of lung parenchyma distorted by nodules with both Ge-imogolites. Ge-imogolite- and asbestos-induced granulomas were rich in thick collagen fibres as shown by the Trichrome blue, Sirius Red and reticulin stainings (Figure 4I-K, O-Q and U-W). Argyrophilic staining of reticulinic fibres revealed organized “smooth” collagen fibres in granulomas and, even in alveoli, a thicker fibre network than in control lungs. Periodic Acid-Schiff (PAS) staining was noted in granuloma macrophages and extracellular spaces of SW and DW lungs, suggesting the accumulation of a glycoprotein-rich material (Figure 4F, L, R and X).

**Figure 5:**
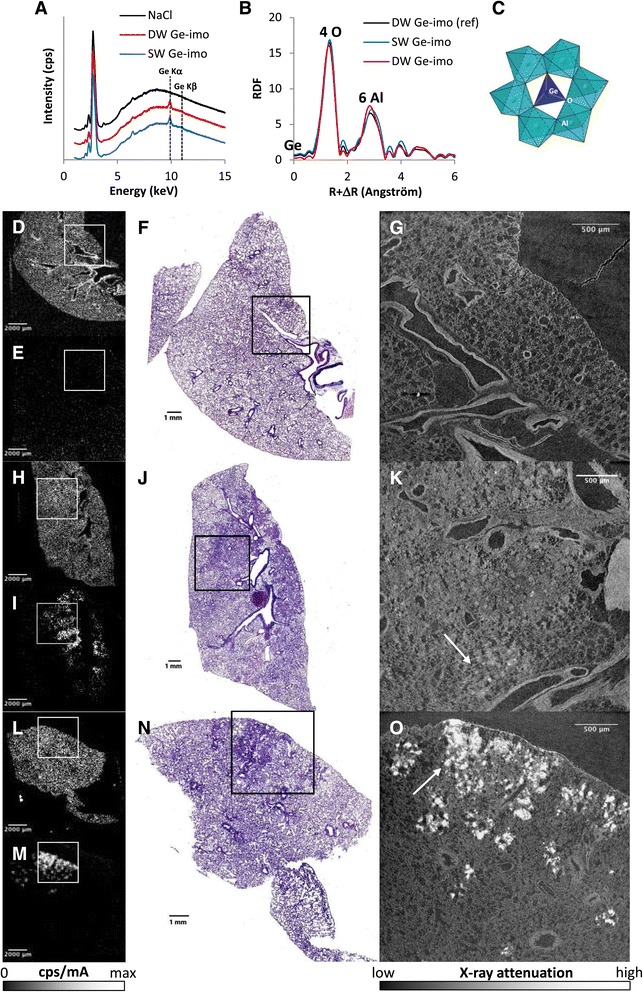
**Ge-imogolites with intact local atomic structure are localized in fibrotic lung zones.** Wistar rats were intra-tracheally instilled with 1 mg SW and DW Ge-imogolites (Ge-imo) or only with their vehicle (NaCl) for control lung. All analyses were performed on paraffin-embedded lung cross sections obtained from animals sacrificed after 60 d. **(A)** micro-XRF sum spectra of lungs from rats instilled with NaCl, DW and SW Ge-imogolites. Sum spectra are extracted from hyperspectral mapping of lung regions (shown in **D-E, H-I, L-M**). Spectra are intentionally shifted along y-axis expressed in arbitrary unit. **(B)** XAS at Ge K-edge: radial distribution functions (RDF) of lungs from rats instilled with SW and DW Ge-imogolites compared to RDF of a DW Ge-imogolite reference sample. **(C)** Theoretical structure of Ge-imogolite showing the coordination environment around Ge: the first atomic shell is attributed to 4 oxygen atoms surrounding the Ge atom at 1,75 Å and the second coordination sphere of Ge corresponds to Ge-Al atomic pairs (theoretical number of 6 Al neighbors around 3,27 Å). Micro-XRF maps (pixel size of 104 μm) showing the distribution of S **(D, H, L)** and Ge **(E, I, M)** in NaCl **(D-E)**, SW **(H-I)** and DW **(L-M)** treated lungs. Pulmonary zones are S positive. Scans of lung upper layer sections stained with H&E from NaCl **(F)**, SW **(J)** and DW **(N)** Ge-imogolite samples. Squares delimitate Ge-rich areas selected for micro-CT. 2D virtual slices extracted from reconstructed micro-CT volume of NaCl **(G)**, SW **(K)** and DW **(O)** Ge-imogolite treated samples. White arrows indicate brilliant areas with high X-ray attenuation (attributed to the presence of Ge-imogolites).

Sixty days pulmonary samples (paraffin-embedded lungs) were investigated to localize Ge-imogolites within the lung. Ge was detected in SW and DW-treated lungs by micro X-ray fluorescence (micro-XRF), but not in control sample (Figure 5A), and was mainly localized in fibrotic alveolar areas (Figure 5D-E, H-I and L-M compared to lung upper layer sections stained with H&E, Figure 5F, J and N). X-ray absorption spectroscopy (XAS) revealed that Ge detected in treated lung exhibited the same local atomic structure as a reference Ge-imogolite (Figure 5B and C). Indeed Ge K-edge EXAFS (extended X-ray absorption fine structure) spectra of SW-, DW-treated lungs and DW Ge-imogolite reference (i.e. with no surface vacancy) were similar. The persistence of intact Ge-imogolite structures in the rat lung is consistent with the poor in vitro solubility of Ge-imogolites (4% dissolution at maximum) previously reported by some of us [21]. Ge-rich lung areas were 3D scanned at high spatial resolution by X-ray computed micro-tomography (micro-CT) and revealed the presence of brilliant zones. The high X-ray attenuation of these voxels (3D-pixels) was attributed to the presence of Ge-imogolites in dense fibrotic zones (Figure 5G, K and O). Since imogolites have a high capacity to adsorb proteins [20] and because we observed glycoprotein-rich material in granulomas (PAS staining), the detection of Ge-imogolites in the same zones suggests that Ge-imogolites can trap proteins and possibly other molecules, leading to the formation of granulomas. Thus, we have shown that a fraction of instilled Ge-imogolites was persistent in the lung, remained chemically and structurally intact and induced a sustained pulmonary inflammation accompanied by a deep alveolar remodeling and accumulation of extra-cellular matrix proteins. This response was more severe, on a mass dose basis, than that induced by crocidolite.

**Figure 6:**
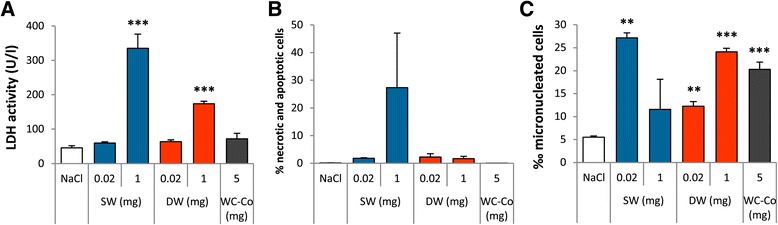
**Ge-imogolites induce genotoxicity in rat lungs.** Wistar rats were intra-tracheally instilled with NaCl (controls), 5 mg WC-Co, 0.02 and 1 mg SW and DW Ge-imogolites. Micronuclei were assessed in isolated AEC-II 3 d after exposure. **(A)** BALF LDH activity, **(B)** % of necrotic and apoptotic cells and **(C)** ‰ micronucleated cells. **P < 0.01 and ***P < 0.001 relative to control rats (Dunnett multiple comparisons test between NaCl and Ge-imogolite-treated rats or t-test between NaCl and WC-Co-treated rats, n = 3, means ± SEM).

In a previous in vitro study on Ge-imogolites, we reported a genotoxic response in dermal cells at non cytotoxic doses [21]. Thus, we assessed the in vivo genotoxic activity of Ge-imogolites by evaluating the formation of micronuclei (MN) in type II pneumocytes (AT-II) isolated from rat lungs 3 d after Ge-imogolite administration. On the basis of the inflammatory data obtained 3 d after exposure (Figure 1), non-inflammatory (0.02 mg/rat) and strongly inflammatory (1 mg/rat) Ge-imogolite doses were selected for this experiment. A separate group of rats was also exposed to the hard metal particles WC-Co (5 mg/rat), a well-known genotoxic compound in rat lung [22]. As expected, in vivo cytotoxicity (LDH activity in BALF) and an increased frequency of micronucleated AT-II (MN ‰) were induced after exposure to WC-Co (Figure 6A and C). The lower dose of SW and DW Ge-imogolites led to a significantly higher MN ‰ than in control rats although it did not induce significant alveolar cytotoxicity/inflammation (Figure 6A and C). The higher dose of DW Ge-imogolites further increased genotoxicity. A lower genotoxic response was recorded after SW Ge-imogolites (1 mg) most probably because of the high proportion of dead cells at this dose (Figure 6B). The high level of necrosis in lungs of 1 mg SW Ge-imogolite-treated rats was also shown by the results of BALF LDH activity (Figure 6A). Since genotoxic effects were already recorded at non-inflammatory doses, our data indicate that MN can, at least in part, result from the primary genotoxic activity of Ge-imogolites, as reported for fibres and CNT [23,24].

These results are consistent with the in vitro genotoxicity data (comet and micronucleus assays) obtained with other Ge-imogolite nanotubes on human fibroblasts [21]. Very recently, low in vitro cytotoxicity and genotoxicity were reported with synthesized Si-imogolites [25]. While this may suggest a different toxicity between Si- and Ge- imogolites, it might also reflect a discrepancy between in vitro and in vivo results. Indeed, the Ge-imogolites used in the present study only induced a weak cytotoxicity on macrophages, epithelial cells and fibroblasts in vitro (not shown).

The fact that SW Ge-imogolites appeared more toxic than DW at the same mass dose is consistent with the higher SW tube number/g (or a higher specific external surface SW Ge-imogolites) compared to DW (see Table 1), suggesting that surface activity drives the toxic response to Ge-imogolites. Mechanistically, several arguments further support the idea that intact Ge-imogolite structures, rather than their elementary components, are responsible for long-term pulmonary effects: (i) intact Ge-imogolite tubes were detected in lung fibrotic areas, (ii) previous studies conducted with Ge metal [26] or Ge oxide [27] did not report similar toxic responses, (iii) contrary to CNT [28], residual catalysts can be excluded as a cause of toxicity since Ge-imogolites are free of such impurities. Furthermore, the overall stronger response to Ge-imogolites and the higher specific external surface of Ge-imogolites compared to crocidolite specific surface area are consistent with the involvement of surface activity in our study.

## Conclusions

In conclusion, we showed for the first time that nanometer-long Ge-imogolites persist in the deep lung and promote inflammation, fibrosis and genotoxicity, indicating that very short HARN can induce severe lung toxicity. Our results highlight the necessity to assess the respiratory hazard of HARN even with a length well below the threshold of 5 μm identified for fibre-induced pulmonary toxicity [1-3].

## Methods

### Ge-imogolites, crocidolite and WC-Co

Ge-imogolites were synthetized, quantified and characterized as described in the Additional file 2. Respirable crocidolite fibres were obtained from the Union Internationale Contre le Cancer (UICC, Geneva, Switzerland) [29]. Hard metal (WC-Co) particles were described previously [22,30]. Crocidolite and WC-Co were first treated at 200°C during 2 h to remove any possible trace of endotoxin. Ge-imogolites localization within treated-lungs and determination of their local structure by X-ray techniques are described in the Additional file 2.

### Animals and treatments

Eight to ten week old female Wistar rats (200–250 g) were obtained from Janvier (St Berthevin, France) and housed in positive pressure air-conditioned units (25°C, 50% relative humidity) on a 12 h light/dark cycle with free access to water and laboratory animal food. Before instillation, animals were anesthetized with a mix of Ketalar (n.v. Warner-Lambert, Zaventem, Belgium) and Rompun (Bayer, Leverkusen, Germany) (respectively 18 and 0.5 mg/rat i.p.). Ge-imogolites, crocidolite and WC-Co were diluted or suspended in NaCl 0.9% and then, serially diluted with the same saline solution. Rats were intra-tracheally instilled into the lungs with 300 μl suspension. Control rats received a corresponding volume of NaCl 0.9%. Animals were sacrificed at selected time points with an overdose of sodium pentobarbital (30 mg/rat given i.p.). The protocols of this investigation were approved by the local committee for animal research at the Université catholique de Louvain, Comité d'Ethique pour l'Expérimentation Animale, Secteur des Sciences de la Santé.

### Bronchoalveolar lavages (BAL) and organ/blood sampling

Blood was collected directly from the heart. BAL was performed by cannulating the trachea and infusing the lungs with 6 ml NaCl 0.9%. For collagen quantification, whole lungs were then perfused with NaCl 0.9%, excised and then placed in 6 ml cold PBS. For Ge quantification (described in the Additional file 2), lungs, spleens, livers, kidneys and brains were directly collected in cold PBS without BAL and perfusion. Organs were homogenized on ice with an Ultra-Turrax T25 (Janke and Kunkel, Brussels, Belgium) and stored at −80°C. Lavages were centrifuged 10 min at 400 *g* (4°C). Cell-free supernatants were used for biochemical measurements and cell pellets were resuspended in PBS. Total BAL cells were counted and pelleted onto glass slides by cytocentrifugation for differentiation by light microscopy after Diff-Quick staining (200 cells counted, Dade Behring AG, Düdingen, Switzerland). Total proteins and lactate dehydrogenase (LDH) activity were assayed on BAL fluids (BALF) as described previously [31].

### Quantification of total lung collagen and BAL cytokines

Collagen deposition was estimated by measuring hydroxyproline content in lung homogenates. Hydroxyproline was assessed by high-pressure liquid chromatography analysis on hydrolyzed lung homogenates (6 N HCl at 108°C during 24 h) as previously described [32]. The following ELISA were performed on BALF according to manufacturer’s instructions (R&D System, Minneapolis, USA): rat DuoSet (for IL-6, IL-1β and TNF-α) and rat Quantikine (for TGF-β1, PDGF-AB and OPN). For determining the influence of Ge-imogolites and crocidolite on the detection of BALF IL-6 and IL-1β in their respective ELISA, standard cytokines were mixed with equivalent BALF volumes from control (NaCl), SW and DW Ge-imogolites and crocidolite-treated rats and standard curves were compared. BALF collected from rats exposed to 0.1 mg SW and 0.2 mg DW Ge-imogolites were selected to assess interferences between Ge-imogolites and ELISA because the highest IL-1β levels were detected at these doses.

### Histology

Paraffin-embedded lung sections were stained with hematoxylin and eosin (H&E, nucleus and cytoplasm staining), Masson’s trichrome blue (collagen staining), Sirius Red (type I collagen staining), reticulin (type III collagen staining), Periodic Acid-Schiff (PAS, glycoprotein staining) for light microscopy examination. For comparison with mXRF and microCT pictures, H&E stained sections were scanned with the Leica SCN400 (Diegem, Belgium). Images were processed with Tissue Image Analysis 2.0 (Leica).

### Ex-vivo micronucleus assay on type II alveolar epithelial cells

Type II alveolar epithelial cells (AEC-II) were collected from lavaged and digested lungs 3 d after intra-tracheal instillation as described in Additional file 2. Briefly, Fc receptor negative cells were incubated 2 d at 37°C and then stained with acridine orange prior to analysis with a fluorescence microscope.

### Statistics

Differences between NaCl and treated groups were evaluated using t tests or one-way analysis of variance, followed by a Dunnett multiple comparisons test, as appropriate. Statistical significance was considered at *P* < 0.05. Data analysis was performed with GraphPad Prism version 3.03 (GraphPad Software, San Diego, USA).

## Additional files

Additional file 1: Figure S1. Characterization of SW and DW Ge-imogolites. (A-C) Calibrated scattered intensity (cm^-1^) as a function of scattering vector q for the R=1.5 (SW) and R=2.5 (DW) Ge-imogolite suspensions. The experimental curves are compared to scattering models in order to determine the tube radius. (A) SW and DW models, (B) SW Ge-imogolite compared to SW model and (C) DW Ge-Imogolite compared to DW model. (D-F) AFM analysis of SW (D, F) and DW (E, F) Ge-imogolites, (D, E) typical AFM pictures, (F) length distributions obtained from the observation of 919 (SW) and 1650 (DW) randomly selected nanotubes. Dotted lines indicate the AFM tip resolution in nm. Additional file 2:
**Methods.**
Additional file 3: Figure S2. Biopersistence of Ge-imogolites in rats after intra-tracheal instillation. Wistar rats were intra-tracheally instilled with 0.02 mg SW and DW Ge-imogolites (6 μg Ge). Ge was measured by ICP-MS after organ or blood mineralization. Ge was quantified in lungs directly after SW (A) or DW (B) Ge-imogolites installation (d 0) and after 15 and 60 d. Non-linear regression (one phase exponential decay) was used to determine SW (R² = 0.883) and DW (R² = 0.824) Ge-imogolite half-lives. *P < 0.05, **P < 0.01 and ***P < 0.001 relative to Ge-imogolite-treated rats at d 0 (Dunnett multiple comparisons test, n = 3-6, means ± SEM). Ge was quantified in blood 3 and 60 d (C) and in organs 60 d (D) after SW or DW Ge-imogolites instillation. Background values measured in control rats (instilled with NaCl) were subtracted (n = 3-6, means ± SEM).

## Abbreviations

AT-II: Type II pneumocytes
BAL: Bronchoalveolar lavage
BALF: BAL fluid
CNT: Carbon nanotube
micro-CT: Computed micro-tomography
DW: Double wall
EXAFS: Extended X-ray absorption fine structure
HARN: High aspect ratio
H&E: Hematoxylin and eosin
ICP-MS: Inductively coupled plasma mass spectrometry
LDH: Lactate dehydrogenase
MN: Micronuclei
MW: Molecular weight
NM: Nanomaterial
PAS: Positive Periodic Acid-Schiff
PBS: Phosphate buffered saline
RDF: Radial distribution function
SW: Single wall
XAS: X-ray absorption spectroscopy
XRF: X-ray fluorescence
