# Supplementary figures and images for: Nanometer-long Ge-imogolite nanotubes cause sustained lung inflammation and fibrosis in rats
Source: Part Fibre Toxicol. 2014 Dec 14;11:67. doi: 10.1186/s12989-014-0067-z (PMC4276264; doi:10.1186/s12989-014-0067-z)

Supplementary figure 1

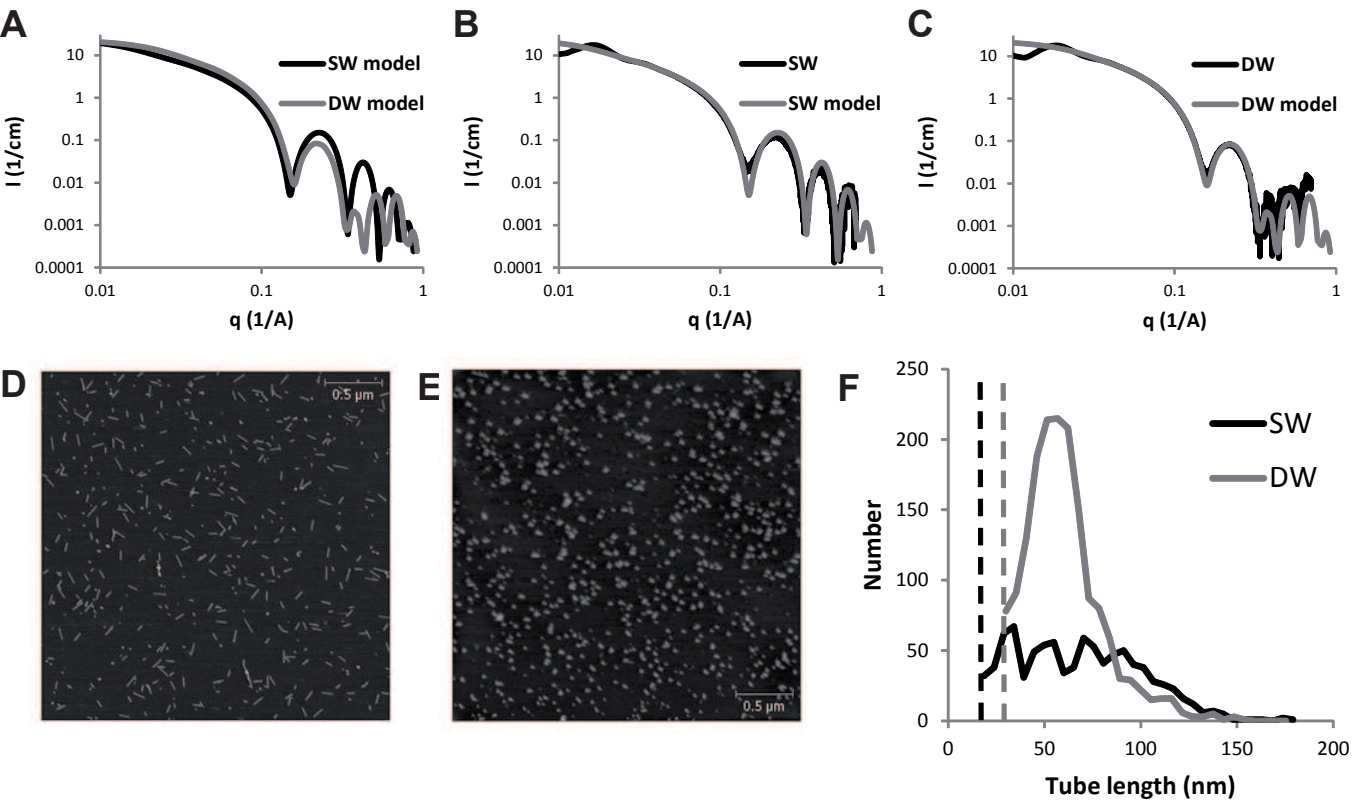

Supplement: Additional file 1: Figure S1. — Characterization of SW and DW Ge-imogolites. (A-C) Calibrated scattered intensity (cm-1) as a function of scattering vector q for the R=1.5 (SW) and R=2.5 (DW) Ge-imogolite suspensions. The experimental curves are compared to scattering models in order to determine the tube radius. (A) SW and DW models, (B) SW Ge-imogolite compared to SW model and (C) DW Ge-Imogolite compared to DW model. (D-F) AFM analysis of SW (D, F) and DW (E, F) Ge-imogolites, (D, E) typical AFM pictures, (F) length distributions obtained from the observation of 919 (SW) and 1650 (DW) randomly selected nanotubes. Dotted lines indicate the AFM tip resolution in nm. [file 12989_2014_67_MOESM1_ESM.pdf]

Supplementary figure 2

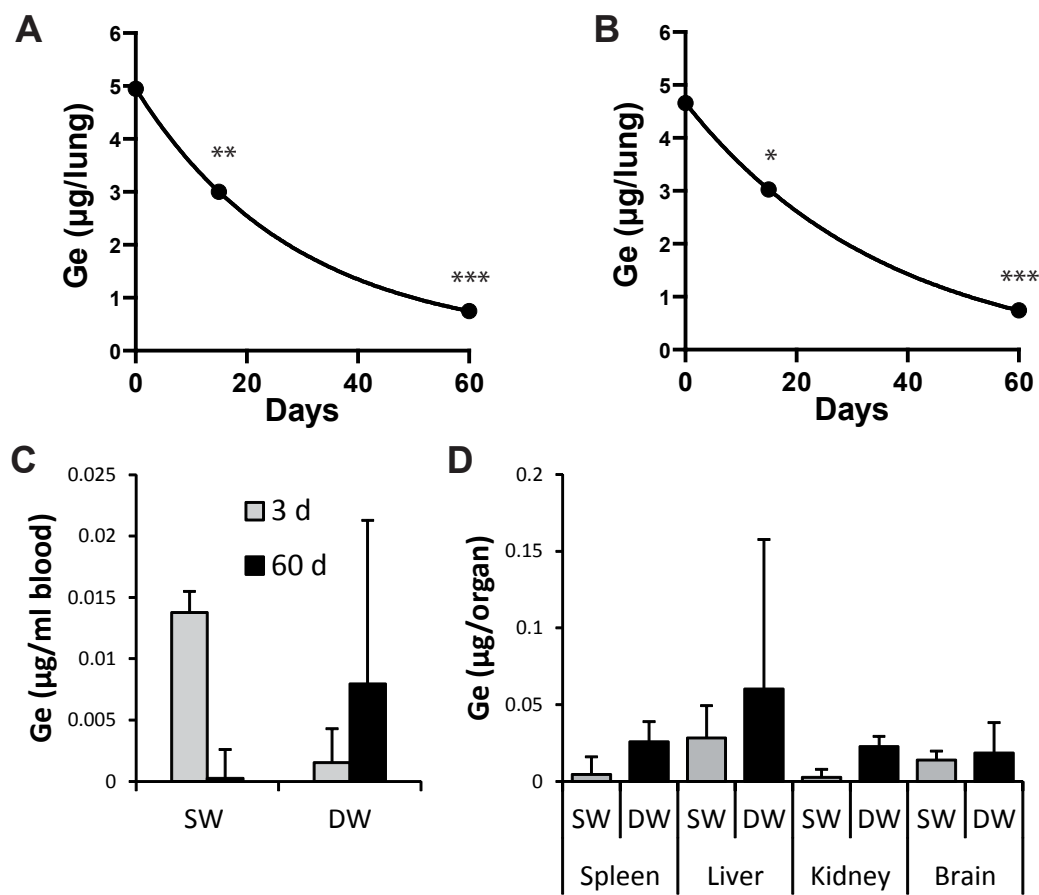

Supplement: Additional file 3: Figure S2. — Biopersistence of Ge-imogolites in rats after intra-tracheal instillation. Wistar rats were intra-tracheally instilled with 0.02 mg SW and DW Ge-imogolites (6 μg Ge). Ge was measured by ICP-MS after organ or blood mineralization. Ge was quantified in lungs directly after SW (A) or DW (B) Ge-imogolites installation (d 0) and after 15 and 60 d. Non-linear regression (one phase exponential decay) was used to determine SW (R² = 0.883) and DW (R² = 0.824) Ge-imogolite half-lives. *P < 0.05, **P < 0.01 and ***P < 0.001 relative to Ge-imogolite-treated rats at d 0 (Dunnett multiple comparisons test, n = 3-6, means ± SEM). Ge was quantified in blood 3 and 60 d (C) and in organs 60 d (D) after SW or DW Ge-imogolites instillation. Background values measured in control rats (instilled with NaCl) were subtracted (n = 3-6, means ± SEM). [file 12989_2014_67_MOESM3_ESM.pdf]
