# Supplementary material for: Nanometer-long Ge-imogolite nanotubes cause sustained lung inflammation and fibrosis in rats
Source: Part Fibre Toxicol. 2014 Dec 14;11:67. doi: 10.1186/s12989-014-0067-z (PMC4276264; doi:10.1186/s12989-014-0067-z)
Supplement: Additional file 2: — Methods. [file 12989_2014_67_MOESM2_ESM.docx]

**Supplementary information**

**Nanometer-long Ge-imogolite nanotubes cause sustained lung inflammation and fibrosis in rats**

Sybille van den Brule, Emilie Beckers, Perrine Chaurand, Wei Liu, Saloua Ibouraadaten, Mihaly Palmai-Pallag, Francine Uwambayinema, Yousof Yakoub, Astrid Avellan, Clément Levard, Vincent Haufroid, Etienne Marbaix, Antoine Thill, Dominique Lison, Jérôme Rose

**Methods**

*Synthesis and preparation of Ge-imogolites*

Ge-imogolites were synthetized by addition of tetraethoxygermanium to an aluminum perchlorate solution with an Al/Ge ratio set to 2. The initial aluminium perchlorate concentration is 0.5 M in fresh MilliQ water. The mixture was slowly hydrolyzed by addition of a NaOH solution (same concentration as the aluminum perchlorate concentration) to reach a hydrolysis ratio ([OH]/[Al]) of 1.5 and 2.5 and form SW and DW Ge-imogolites, respectively [1]. The obtained solution was stirred overnight and introduced into a thermostated reactor at 90°C for five days. Each sample was dialyzed against ultrapure water, using a 8-10 kDa membrane in order to remove residual salts and excess alcohol. Obtained Ge-imogolite stock suspensions were filter-sterilized (0.22 µm) before use. Ge quantification by inductively coupled plasma mass spectrometry (ICP-MS, 7500ce, Agilent, USA) before and after filtering showed no difference in concentration.

*Quantification of Ge*

Ge was quantified in Ge-imogolite stock suspensions diluted with 0.05 N HNO_3_ and mineralized organs and blood (after rat instillation) by ICP-MS. Two ml organ homogenates or blood were mineralized in acid (5 ml HNO_3_ 65 % and 0.5 ml HCl 30 %) during 24 h at 60°C. After total evaporation, residues were suspended in 5 ml 0.05 N HNO_3_. The Ge content was then determined by ICP-MS using collision cell in helium mode and Rh as internal standard.

*Small Angles X-ray Scattering (SAXS)*

The two imogolite samples were analyzed by SAXS. This technique allows the determination of the shape of the nanotubes in suspension. The set up includes a rotating anode and collimating optics providing a monochromatic X-ray beam (λ=0.1548 nm) of 2x2 mm^2^ at the sample position with a total incident flux of 8.10^7^ photons/s. The transmitted flux is measured continuously with a photodiode placed on the beam stop. A MAR research X-ray sensitive 300 mm plate detector is placed after the output window of the vacuum chamber. The scattering vector q is defined as q=k_d_-k_i_ (the wave vectors of the incident and scattered beams) and has a modulus of q = 4π/λ sin(θ) where λ is the incident wavelength and 2θ is the scattering angle. The distance from the sample to the detector is determined using a tetradecanol lamellar phase having its first order peak at q=0.1581Å^-1^. A ratio q_max_/q_min_ of 34 is reached with q_max_=0.5 Å^-1^ and q_min_=0.015 Å^-1^. The samples were introduced in 0.13 cm Kapton capillaries (Microlumen). The counting time is 3600 s and the signal is corrected for background. Standard procedures are applied to obtain the scattered intensity in cm^-1^ as a function of scattering vector q [2].

Supplementary figures 1 B and C shows the SAXS experimental curves obtained for Ge-imogolites prepared with R=1.5 and 2.5. As already observed, the curve shape changes from SW to DW when the hydrolysis ratio increases from 1.5 to 2.5 [1]. Each SAXS curve is adjusted with a scattering model for homogeneous cylinders (supplementary figure 1A, adapted from Amara et al, 2013 [3]). The electronic density of the nanotube wall is taken to be 0.91 e^-^.Å^-3^ and the water electronic density considered homogeneous and equal to 0.334 e^-^.Å^-3^. SAXS simulations reveal that the experimental signal is really nicely explained by the scattering of a suspension of well-defined SW and DW nanotubes (See table 1).

*Tube length distribution by atomic force microscopy (AFM)*

Tube length of SW and DW Ge-imogolites stock solutions were measured using AFM (Innova Atomic Force Microscope, Bruker). Typical AFM images (512 x 512 pixels) were recorded in tapping mode with a pixel size of 5.85 nm. A diluted drop of Ge-imogolite stock solution was deposited on a cleaved mica sheet and then dried before measurement. The tip resolution of SW and DW Ge-imogolite images is 18 nm and 30 nm, respectively. AFM image analysis was performed with the open source ImageJ software. The plugin called “Tubeness” for ImageJ software was applied on raw image to improve tube separation [4]. Tube length distribution was obtained after threshold and labelling steps. Lengths were estimated by measuring the Feret’s diameter of the labeled tube.

*Spatial distribution of Ge-imogolites within the lung: coupled 2D and 3D X-ray imaging*

Chemical mapping of paraffin-embedded lungs (cross section) was performed by X-ray fluorescence micro-spectroscopy (micro-XRF). Measurements were carried out on a microscope (XGT^7000^, Horiba Jobin Yvon) equipped with a focused X-ray source (incident beam spot of 100 μm produced with a Rh-tube, accelerating voltage of 30 kV and current of 1 mA) and an energy dispersive X-ray (EDX) spectrometer. As the incident X-ray beam penetrates through the sample, the obtained element maps are 2D projections of the 3D analyzed sample. Elements from Mg to U can be detected with a sensitivity range from about 50 mg/kg to few % mass depending on the atomic number of the element and the nature of the probed matrix.

Coupled 3D imaging was performed by X-ray computed micro-tomography (micro-CT) without requirement of extra sample preparation. Micro-CT is a three-dimensional and non-destructive imaging technique that is based on the 3D computed reconstruction of a sample from the 2D radiographic projections acquired at different angles around its axis of rotation. For each sample, 1601 projection images with an angle step of 0.225° (through the 360° rotation) and an exposure time of 5 s each were recorded with the Zeiss Xradia Micro-XCT-400 system. The beam energy was set to 41 kV (accelerating voltage of W X-ray tube) and the current (i.e. beam intensity) to 219 μA.

The 4X magnification optical objective was selected, leading to a spatial resolution around 7 μm for a field of view (FOV) of 4.24 mm. The selected FOV was centered in Ge-rich area of paraffin-embedded treated-lung identified by micro-XRF. The output of micro-CT scanning is a reconstructed 3D volume of local X-ray attenuation coefficients, which depend on both material composition and density. Reconstruction was done using a Zeiss Xradia software, the obtained 3D-image dataset was composed of 1601 contiguous grayscale images where each slice image contained 1000x1000 voxels (volume element or 3D pixels), with an effective size of 4,24 μm and an intensity (or grayscale value, GSV) proportional to the X-ray absorption by the sample. Thus darker voxels correspond to low-density phases (ex. lung tissue) and brighter voxels are associated to high-density phases (ex. Ge-imogolites).

*Ge-imogolite local structure within the lung: X-ray absorption spectroscopy at Ge K-edge*

XAS experiments were performed at the European Synchrotron Radiation Facility (ESRF-Grenoble, France) on the FAME beamline (BM30b) with a Si(220) monochromator[5]. Focused incident beam (spot size of 300x200 μm) was centered on Ge-rich areas previously scanned by micro-XRF and micro-CT. During scan acquisition, samples are cooled to about 10K in a He-cryostat to decrease thermal motion. Multiple scans were collected for each sample in fluorescence mode above the Ge K-edge (11103 eV). EXAFS (extended X-ray fine structure) data were obtained after standard procedures using the IFEFFIT software package[6].

*Ex-vivo micronucleus assay on type II alveolar epithelial cells*

BAL was performed with 6 ml solution I (NaCl 8.18 g/l, KCl 0.373 g/l, NaH_2_PO_4_.2H_2_O 0.075 g/l, NaH_2_PO_4_ 0.287 g/l, HEPES 2.38 g/l, dextrose 1.08 g/l) and lungs were then perfused with 20 ml solution I and lavaged 8 times with 6 ml EGTA 0.2 mM (pH 7.4). Lungs were lavaged once with 6 ml elastase (Sigma, St Louis, USA, 1.2 U/ml solution II, i.e. NaCl 8.18 g/l, KCl 0.373 g/l, NaH_2_PO_4_.2H_2_O 0.075 g/l, NaH_2_PO_4_ 0.287 g/l, HEPES 2.38 g/l, dextrose 1.08 g/l, CaCl_2_.H_2_O 0.294 g/l, MgSO_4_ 0.156 g/l, MgSO_4_.7H_2_O 0.32 g/l) and injected with 15 ml elastase pre-warmed at 37°C. Lungs were transferred in 50 ml tubes filled with 20 ml elastase solution and incubated 30 min at 37°C to release type II alveolar epithelial cells (AEC-II). The digested tissue was then minced with scissors and 5 ml DNase (Worthington Biochemical Corporation, Lakewood, USA, 0.2 mg/ml in solution II) and 5 ml fetal bovine serum (FBS) low IgG (Invitrogen) were added. Cell suspensions were shaken for 15 min and filtered to obtain individual and/or clumps of a few cells. Cells were centrifuged 10 min at 400 *g* (4°C), resuspended in Dulbecco’s Modified Eagle’s Medium (DMEM, Invitrogen) high glucose HEPES (Invitrogen) supplemented with 1 % antibiotic-antimycotic (aa, Invitrogen) and incubated in rat IgG (Sigma) pre-coated plates during 1 h at 37°C to remove cells bearing Fc receptors. Non-adherent cells were then collected, centrifuged 10 min at 400 *g* (4°C), resuspended in Waymouth’s medium (Invitrogen) supplemented with 10 % FBS and 1 % aa and counted using the Trypan blue exclusion technique. 500.000 cells/well were plated onto chamber slides (4 well glass slides coated with fibronectine, BD Biosciences, Erembodegem, Belgium) and incubated at 37°C. After 2 d, AEC-II were fixed in 100% methanol (20 min) and stained with acridine orange (0.012 % in phosphate buffer) prior to analysis with a Zeiss Axioscop fluorescence microscope (magnification 400×). Multiple culture wells were analyzed for each animal. In total, 2000 AEC-II cells per rat were evaluated for the presence of micronuclei. Apoptotic cells were visually identified as cells with chromatin condensation or nuclear fragmentation into smaller nuclear bodies within an intact cytoplasm. Necrotic cells exhibited a pale cytoplasm with numerous vacuoles and damaged cytoplasmic membrane.

**Supplementary figure legends**

**Supplementary figure 1: Characterization of SW and DW Ge-imogolites**

(a-c) Calibrated scattered intensity (cm^-1^) as a function of scattering vector q for the R=1.5 (SW) and R=2.5 (DW) Ge-imogolite suspensions. The experimental curves are compared to scattering models in order to determine the tube radius. (A) SW and DW models, (B) SW Ge-imogolite compared to SW model and (C) DW Ge-Imogolite compared to DW model. (D-F) AFM analysis of SW (D, F) and DW (E, F) Ge-imogolites, (D, E) typical AFM pictures, (F) length distributions obtained from the observation of 919 (SW) and 1650 (DW) randomly selected nanotubes. Dotted lines indicate the AFM tip resolution in nm.

**Supplementary figure 2: Biopersistence of Ge-imogolites in rats after intra-tracheal instillation**

Wistar rats were intra-tracheally instilled with 0.02 mg SW and DW Ge-imogolites (6 µg Ge). Ge was measured by ICP-MS after organ or blood mineralization. Ge was quantified in lungs directly after SW (A) or DW (B) Ge-imogolites installation (d 0) and after 15 and 60 d. Non-linear regression (one phase exponential decay) was used to determine SW (R² = 0.883) and DW (R² = 0.824) Ge-imogolite half-lives. * P < 0.05, ** P < 0.01 and *** P < 0.001 relative to Ge-imogolite-treated rats at d 0 (Dunnett multiple comparisons test, n = 3-6, means ± SEM). Ge was quantified in blood 3 and 60 d (C) and in organs 60 d (D) after SW or DW Ge-imogolites instillation. Background values measured in control rats (instilled with NaCl) were subtracted (n = 3-6, means ± SEM).

**Reference List**

1. Thill A, Maillet P, Guiose B, Spalla O, Belloni L, Chaurand P, Auffan M, Olivi L, Rose J: **Physico-chemical control over the single- or double-wall structure of aluminogermanate imogolite-like nanotubes.** *J Am Chem Soc* 2012, **134:**3780-3786.

2. Lindner P, Zemb T: *Neutrons, X-rays and Light: Scattering Methods Applied to Soft Condensed Matter.* North-Holland; 2002.

3. Amara MS, Paineau E, Bacia-Verloop M, Krapf ME, Davidson P, Belloni L, Levard C, Rose J, Launois P, Thill A: **Single-step formation of micron long (OH)3Al2O3Ge(OH) imogolite-like nanotubes.** *Chem Comm* 2013, **49:**11284-11286.

4. Sato Y, Nakajima S, Shiraga N, Atsumi H, Yoshida S, Koller T, Gerig G, Kikinis R: **Three-dimensional multi-scale line filter for segmentation and visualization of curvilinear structures in medical images.** *Med Image Anal* 1998, **2:**143-168.

5. Proux O, Nassif V, Prat A, Ulrich O, Lahera E, Biquard X, Menthonnex JJ, Hazemann JL: **Feedback system of a liquid-nitrogen-cooled double-crystal monochromator: design and performances.** *J Synchrotron Radiat* 2006, **13:**59-68.

6. Ravel B, Newville M: **ATHENA, ARTEMIS, HEPHAESTUS: data analysis for X-ray absorption spectroscopy using IFEFFIT.** *J Synchrotron Radiat* 2005, **12:**537-541.
